# Monitoring Protein Dynamics in Protein *O*-Mannosyltransferase Mutants In Vivo by Tandem Fluorescent Protein Timers

**DOI:** 10.3390/molecules23102622

**Published:** 2018-10-12

**Authors:** Joan Castells-Ballester, Ewa Zatorska, Matthias Meurer, Patrick Neubert, Anke Metschies, Michael Knop, Sabine Strahl

**Affiliations:** 1 Centre for Organismal Studies (COS), Heidelberg University, 69120 Heidelberg, Germany; joan.castells-ballester@cos.uni-heidelberg.de (J.C.B.); ewa.zatorska@cos.uni-heidelberg.de (E.Z.); patrick.neubert@cos.uni-heidelberg.de (P.N.); anke.metschies@cos.uni-heidelberg.de (A.M.); 2 Zentrum für Molekulare Biologie der Universität Heidelberg (ZMBH), DKFZ-ZMBH Alliance, Heidelberg University, 69120 Heidelberg, Germany; m.meurer@zmbh.uni-heidelberg.de (M.M.); m.knop@zmbh.uni-heidelberg.de (M.K.); 3 Deutsches Krebsforschungszentrum (DKFZ), DKFZ-ZMBH Alliance, 69120 Heidelberg, Germany

**Keywords:** glycosylation, mannosyltransferase, fluorescent protein timers, secretory pathway, *O*-mannosyl glycans, protein turnover, *Saccharomyces cerevisiae*, PMT1, PMT2, PMT4, yeast

## Abstract

For proteins entering the secretory pathway, a major factor contributing to maturation and homeostasis is glycosylation. One relevant type of protein glycosylation is *O*-mannosylation, which is essential and evolutionarily-conserved in fungi, animals, and humans. Our recent proteome-wide study in the eukaryotic model organism *Saccharomyces cerevisiae* revealed that more than 26% of all proteins entering the secretory pathway receive *O*-mannosyl glycans. In a first attempt to understand the impact of *O*-mannosylation on these proteins, we took advantage of a tandem fluorescent timer (tFT) reporter to monitor different aspects of protein dynamics. We analyzed tFT-reporter fusions of 137 unique *O*-mannosylated proteins, mainly of the secretory pathway and the plasma membrane, in mutants lacking the major protein *O*-mannosyltransferases Pmt1, Pmt2, or Pmt4. In these three *pmt*Δ mutants, a total of 39 individual proteins were clearly affected, and Pmt-specific substrate proteins could be identified. We observed that *O*-mannosylation may cause both enhanced and diminished protein abundance and/or stability when compromised, and verified our findings on the examples of Axl2-tFT and Kre6-tFT fusion proteins. The identified target proteins are a valuable resource towards unraveling the multiple functions of *O*-mannosylation at the molecular level.

## 1. Introduction

*O*-Mannosylation is an evolutionarily conserved protein modification that was first described in the yeast *Saccharomyces cerevisiae* [1]. Very recently, different families of protein *O*-mannosyltransferases have been identified, and it has become obvious that *O*-mannosylation is much more diverse than originally thought [2]. Here, we are focusing on *O*-mannosylation based on PMT-family Protein *O*-MannosylTransferases. This essential protein modification is conserved among fungi, animals, and human [3].

PMTs initiate *O*-mannosylation in the endoplasmic reticulum (ER) transferring mannose from dolichol phosphate-activated mannose to serine and threonine residues of their protein substrates [3]. In baker’s yeast, the PMT-family comprises seven members (Pmt1 to Pmt7), among which Pmt1-Pmt2 heteromeric and Pmt4 homomeric complexes are the most potent transferases in vivo [4]. These polytopic ER membrane enzymes act on their substrate proteins during and/or after translocation of those into the ER, and show different selectivity towards acceptor polypeptides [4]. Several substrate proteins are mainly mannosylated by Pmt1-Pmt2, others by Pmt4. In addition, there are examples, such as Ccw5, in which Pmt1-Pmt2 and Pmt4 complexes act on the same protein, but mannosylate distinct regions [5]. Usually, canonical target proteins are highly *O*-mannosylated, and the mannosyl chains are clustered in serine/threonine-rich regions. Signals determining *O*-mannosylation of substrate proteins and selectivity of the PMT-family members are not yet understood. *O*-Mannosylated proteins can leave the ER and enter the Golgi apparatus once they are properly folded. The protein-bound mannoses can be further elongated by α-1,2- and α-1,3-mannosyltransferases in the Golgi apparatus with GDP-mannose as carbohydrate donor [6]. In this way, short linear oligomannose chains are formed. The recently established *S. cerevisiae O*-mannose glycoproteome showed that at least 26% of the proteins that are targeted to the secretory pathway receive *O*-mannosyl glycans [7]. 

Baker’s yeast is an ideal eukaryotic model to study the functional impact of PMT-based *O*-mannosylation. The characterization of viable single (e.g., *pmt1*Δ, *pmt2*Δ or *pmt4*Δ) and conditional lethal multiple *pmt*Δ (e.g., *pmt2pmt4*Δ) mutants revealed that *O*-mannosylation is important for the formation and maintenance of a functional cell wall, which is a vital structure for yeasts and other fungi [8]. Genome-wide studies further showed that biosynthetic pathways of certain cell wall components, as well as signaling pathways that counteract cell wall defects, are crucial for cell growth when *O*-mannosylation becomes limiting [9,10]. In addition, a signaling pathway that counteracts unbalanced ER protein homeostasis, namely the unfolded protein response, becomes indispensable for cell growth when *O*-mannosylation is impaired, suggesting a role of this modification for proper protein maturation, stability, and/or localization [9,10]. In agreement, *O*-mannosyl glycans are known to play crucial roles in maintaining the stability of certain canonical substrates of PMTs. For example, abrogating *O*-mannosylation by Pmt1, Pmt2, or Pmt4 impacts the maturation and significantly reduces the stability of the plasma membrane sensors of cell wall integrity: Wsc1, Wsc2, Mid2, and Mtl1 [11,12]. Similar effects were observed for Sec20, a tSNARE involved in retrograde vesicle trafficking, which displays maturation defects and decreased stability in *pmt*Δ mutants of the human pathogenic yeast *Candida albicans* [13]. Pmt1 and Pmt2 also play a role in ER protein quality control, which broadly monitors the folding state of secretory proteins and triggers their degradation in case of error [14]. In this scenario, *O*-mannosyl glycans can increase the solubility of misfolded proteins, and thereby support their exit from the ER. Depending on the nature of the protein model studied, degradation by either the ER-associated degradation pathway or in the vacuole, as well as secretion out of the cell, have been reported [15,16,17,18,19]. *O*-Mannosyl glycans have even been suggested as molecular timer abrogating futile folding cycles of polypeptide chains in the ER [20]. The diverse effects of *O*-mannosyl glycans on both canonical acceptor proteins and in protein quality control so far have been studied for a very limited number of proteins [4].

We recently described the yeast *O*-mannose glycoproteome and identified 293 *O*-mannosylated putative PMT-target proteins [7]. In addition to cell wall proteins, many proteins of the secretory pathway and the plasma membrane have been identified as targets of *O*-mannosylation for the first time. In a first attempt to understand the impact of *O*-mannosylation on these proteins, it is imperative to know whether (and which of) the substrate proteins are affected in terms of their stability and localization in the absence of *O*-mannosylation. In this study, we used fusions to the tandem fluorescent timer reporter to monitor protein dynamics of 137 unique *O*-mannosylated proteins in *pmt1*Δ, *pmt2*Δ, and *pmt4*Δ mutants in vivo. This reporter functions as a fluorescent timer protein, and by this it accounts for protein stability. Since fluorescent proteins are affected also by the physico-chemical environment, they simultaneously also serve as sensors for the altered localization of proteins [21,22,23].

## 2. Results

### 2.1. Large Scale Analysis of the Impact of O-Mannosylation on Protein Stability Using Tandem Fluorescent Protein Timers

**Figure 1:**
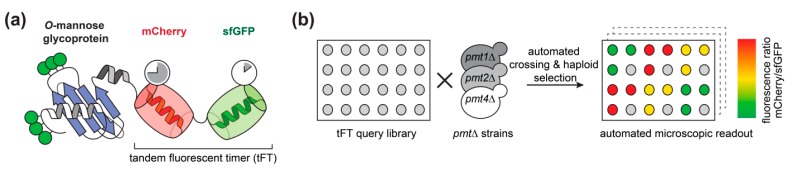
Tandem fluorescent protein timer (tFT) screening. (**a**) Representation of a C-terminal tFT-fusion protein analyzed. The slow maturing mCherry and the fast maturing sfGFP are fused in tandem to the C-terminus of *O*-mannosylated proteins of interest. (**b**) Workflow of the screening of the selected fusion proteins. In brief, 137 individual tFT fusions of the tFT library established by Khmelinskii and coworkers [21] were selected based on the presence of *O*-mannosyl glycans on these proteins [7]. The 137 tFT query strains were crossed with *pmt1*Δ (MLY201), *pmt2*Δ (MLY202), or *pmt4*Δ (MLY204) mutants using synthetic genetic array methodology [24]. Haploid yeast strains carrying both genetic modifications (tFT-fusion and *pmt* deletion) were selected. mCherry/sfGFP ratio was calculated for each protein and used for comparison between wild-type and mutants.

We used the tandem fluorescent protein timer (tFT) approach to address the impact of *O*-mannosylation on the stability of secretory and membrane proteins in living cells. As demonstrated recently by mass spectrometry ([7]; Supplemental Table S1), 137 candidate proteins carrying *O*-mannosyl glycans were selected from a genome-wide library of yeast strains each expressing a different C-terminally tagged tFT-fusion protein [21]. The tFT tag is composed of two fluorescent proteins, mCherry and superfolder GFP (sfGFP), whose fluorophores have distinct maturation kinetics (Figure 1a, [21]). The fluorophore of sfGFP matures rapidly and becomes fluorescent shortly after protein translation is completed. In contrast, the fluorophore of mCherry matures slowly and the protein takes much longer to become fluorescent. The fluorescence intensities of the two proteins can be monitored and quantified independently of each other in vivo. The difference in green fluorescence by sfGFP is therefore indicative for changes in protein abundance. The difference in mCherry fluorescence compared to sfGFP fluorescence intensity, on the other hand, allows us to measure differences in steady-state protein stability: a decrease of the intensity ratio indicates an increase of the degradation rate of the tFT-fusion protein, and vice versa [21]. In addition, both proteins exhibit different pKa values and protease sensitivity. Thus, in case the tFT reporter is facing the lumen, it can be expected that it might also report alterations of the environment, e.g., neutral pH in the ER and Golgi versus acidic pH in the vacuole.

Putting our main focus on *O*-mannosylated proteins of the endomembrane system, the vacuole and the plasma membrane, we introduced *pmt1*Δ*, *pmt2**Δ and**pmt**4Δ deletion alleles into the selected subset of the tFT library strains using high-throughput genetic crosses ([24]; Supplemental Table S1). Three replicates of each cross and an untagged control strain (to monitor background fluorescence) were arranged next to each other on synthetic complete medium lacking leucine. The mCherry and sfGFP fluorescence intensities of the final yeast colonies were recorded after growth at 30 °C for 24 h using a fluorescent plate reader (Figure 1b).

**Figure 2:**
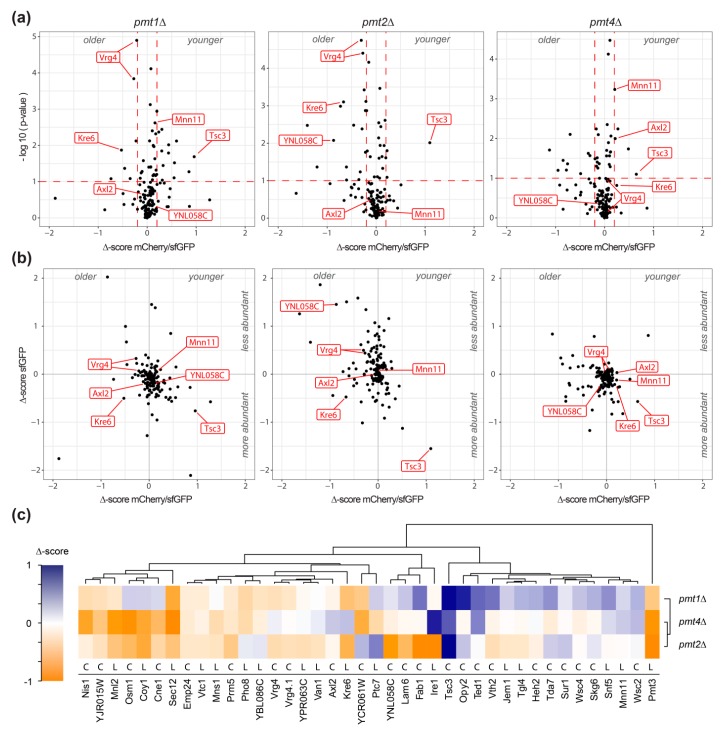
Identification of proteins affected in *pmt1*Δ, *pmt2*Δ and *pmt4*Δ deletion mutants. (**a**) Volcano plots illustrating changes in tFT-fusion protein stability in the indicated mutant strains with regard to statistical significance of data as inferred from variance analysis. Plots show Δ-scores mCherry/sfGFP for changes in protein stability on the x-axis and the negative logarithm of *p*-values on the y-axis. Data were subset for relevance based on thresholds as indicated by red dashed lines (*p*-value < 0.1 and net Δ-score > 0.2). (**b**) Correlation of Δ-scores mCherry/sfGFP, as a measure of tFT-fusion protein stability and turnover (x-axis), and Δ-scores sfGFP, as a measure of change in protein abundance (y-axis). (**a**,**b**) Data referring to proteins further analyzed or discussed in this study are labeled in red. The example of Vrg4-tFT shows, that even minor effects could be reproducibly detected. (**c**) Heatmap with hierarchical clustering of Δ-scores mCherry/sfGFP from a subset of data that passes the thresholds of significance in at least one of the indicated mutant strains (*p*-value < 0.1 and net Δ-score > 0.2). Cytosolic and luminal orientation of the tFT reporter is indicated with C and L, respectively.

The effect of *pmt1*Δ, *pmt2*Δ, and *pmt*4Δ deletion on each of the 137 unique tFT-fusion proteins was quantified as Δ-score, as detailed in Materials and Methods. sfGFP and mCherry/sfGFP intensity Δ-scores are indicative for changes in protein abundance and stability, respectively. Negative mCherry/sfGFP Δ-score values indicate stabilization, while positive values show destabilization of the tFT-fusion protein in the mutant compared to the wild-type. Further, for fusion proteins with the tFT-reporter positioned in the lumen of the secretory pathway or the vacuole, changes in Δ-score values might also point to differences in localization. As shown in Figure 2a,b, the majority of the analyzed proteins were not or only marginally affected in the three mutants (Supplemental Table S1; Supplemental Figure S1). For some proteins, however, significant changes of the Δ-score values could be detected (Figure 2a,b; Supplemental Table S1). In the three *pmt*Δ mutants, a total of 39 individual proteins were clearly influenced. Among those, the tFT-fusion proteins Pmt3, Tsc3, Kre6, Opy2, Vth2, Ted1, Fab1, Lam6, YNL058c, Coy1, Osm1, YCR061w, Sec12, Nis1, and Mnl2 showed the strongest changes (Supplemental Table S1; net Δ-score > 0.5; *p*-value < 0.1). According to TOPCONS [25], C-terminal tFT reporter of at least 59% of the identified proteins is oriented towards the cytosol (Figure 2c). Accordingly, their Δ-scores are indicative of relevant changes in protein abundance and/or stability. We also analyzed the localization of genuine representatives with different C-terminal orientation of the reporter (e.g., Axl2, Coy1, Kre6, Mnn11, Osm1, Sec12, Ted1, Tsc3, Vrg4, Wsc2) using live fluorescence microscopy. No major differences in localization could be observed between wild-type and mutant for any of those proteins (see below and data not shown). Thus, it is highly likely that for the majority of the identified tFT proteins, abundance, and/or stability, rather than localization, are affected.

Hierarchical clustering groups selected tFT-fusion proteins, which are affected similarly by a lack of Pmt1, Pmt2, or Pmt4, and highlights differences in response to the absence of particular Pmts (Figure 2c). For instance, Pmt3 and Sec12 are stabilized in the three mutants, whereas Tsc3 and Wsc2 are destabilized. In agreement, an increase in the abundance of the PMT-family member Pmt3, as well as aberrant maturation of the plasma membrane sensor protein Wsc2, have been shown in *pmt*Δ mutants at steady state in the past by Western blot [11,26,27,28]. Other tFT-fusion proteins, however, show distinct effects; for instance, Opy2 is destabilized in *pmt1*Δ and *pmt2*Δ, but not *pmt4*Δ, whereas for Axl2 (see below), the opposite effect holds true.

In summary, using the tFT technology, we identified several glycoproteins that are affected most likely in abundance and/or stability upon decrease of *O*-mannosylation. Consistent with the manifold effects of *O*-mannosyl glycans described so far, protein stabilization as well as destabilization could be observed.

### 2.2. Validation of the Screening Results on the Example of Axl2

To further address the validity of our findings, we chose the well-characterized type I plasma membrane protein Axl2 as an example. Axl2 was among the proteins with the strongest ratio change destabilized proteins in mutant pmt*4*Δ (Figure 2a–c). Axl2 is required for axial budding in haploid cells [29]. The glycoprotein has been experimentally confirmed as a canonical Pmt4-specific substrate which is less stable in absence of *O*-mannosylation [30]. In agreement, our screen identified the Axl2-tFT protein to be destabilized in *pmt4*Δ but not in pmt1Δ and pmt2Δ mutants (Figure 2c). We created an independent *pmt4*Δ knock out strain expressing Axl2-tFT and confirmed the destabilization of the tFT-fusion protein also under conditions of logarithmic growth using fluorescence flow cytometry (Table 1).

**Figure 3:**
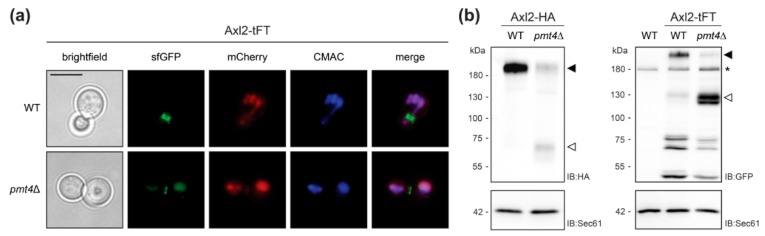
Analyses of Axl2-tFT protein. (**a**) Live fluorescence microscopy of both wild-type (WT; WT Axl2-tFT) and *pmt4*Δ (EZY107) cells expressing the Axl2-tFT. Prior to imaging, cells were stained with the vacuolar vital dye 7-amino-4-chloromethylcoumarin (CMAC). Scale bar, 5 µm. (**b**) Membranes (equivalent to 1 OD_600_ units of yeast cells) from wild-type and *pmt4*Δ cells expressing Axl2 C-terminally tagged with either HA or tFT (strains MGY69, MGY72, WT Axl2-tFT, and EZY107), were resolved on 8% polyacrylamide gels and subjected to Western blot analysis using anti-HA and anti-GFP antibodies, respectively. Full length form of tagged Axl2 (black arrows) is less abundant in *pmt4*Δ than in corresponding wild-type cells. In the cells lacking Pmt4, C-terminal proteolytic fragments of the protein (white arrows) are detected. Differences in the apparent molecular masses of the full-length forms and proteolytic fragments observed for Axl2-HA and Axl2-tFT, respectively, correspond to the calculated mass difference (−60 kDa) of the tags. Asterisks indicate an unrelated cross-reactive band, present also in the membranes isolated from wild-type cells without tFT-fusion protein (YMaM330). As a loading control, the blot was subsequently incubated with anti-Sec61 antibody. Experiments have been replicated three times; representative results are shown.

As described for the wild-type protein, Axl2-tFT marks the presumptive bud site and is found in ring structures at the mother-bud neck in wild-type cells (Figure 3a, [29,30]). In the mutant *pmt4*Δ, the Axl2-tFT protein is largely localized to the vacuole and shows a similar degradation pattern to that previously described for a functional HA-tagged version of the protein (Figure 3b, [30]).

Our data confirm that the tFT readout allows tracking the impact of *O*-mannosylation on the stability of glycoproteins and thereby even PMT-specific substrates can be identified.

### 2.3. Defects in O-Mannosylation Result in Protein Stabilization

Interestingly, our screening revealed many proteins to be stabilized when *O*-mannosylation is decreased (supplemental Table S1; Figure 2a–c). This finding is particularly interesting in view of the suggested role of *O*-mannosylation by Pmt1 and Pmt2 in the turnover of several misfolded protein models [14].

To further examine this issue, we created independent *pmt*Δ mutants expressing the most stabilized tFT-fusion proteins identified with a *p*-value < 0.015 in *pmt1*Δ or *pmt2*Δ (Supplemental Table S1), and analyzed protein stability by fluorescence flow cytometry. Among the selected tFT-fusions, stabilization of Kre6, Vrg4 and YNL058c could also be confirmed under conditions of logarithmic growth (Table 1).

To rule out effects of *pmt*Δ strains leading to protein stabilization that are not related to protein turnover, only strains with sfGFP/mCherry and sfGFP intensity Δ-score values that indicate increased stability and abundance, respectively, were considered. The Kre6-tFT fusion protein meet all the requirements suggesting impaired degradation, and showed the biggest change of both parameters compared to other tFT-fusions in mutants *pmt1*Δ as well as *pmt2*Δ (Figure 2a,b).

**Figure 4:**
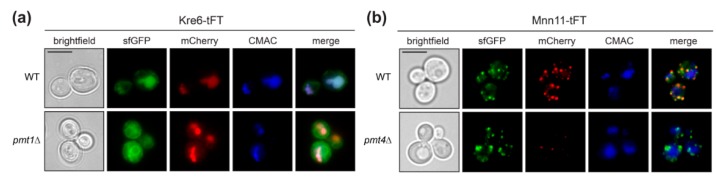
Localization of Kre6-tFT protein. Live fluorescence microscopy of wild-type (WT Kre6-tFT) and *pmt1*Δ (EZY91) cells expressing the Kre6-tFT (**a**), and of wild-type (WT Mnn11-tFT) and *pmt4*Δ (EZY109) cells expressing the Mnn11-tFT (**b**). (**a**) In wild-type and *pmt1*Δ cells, the type II transmembrane protein Kre6-tFT (Figure 5a) is present in the ER, but mainly in the vacuole. (**b**) Localization of another type II transmembrane protein, Mnn11-tFT (Figure 5a), to the Golgi is not affected by the C-terminal tFT reporter. Lower abundance of old Mnn11-tFT (mCherry) in *pmt4*Δ cells when compared to wild-type strain confirms the destabilization of this protein observed upon decreased *O*-mannosylation (Figure 2c). Prior to imaging, cells were stained with the vacuolar vital dye CMAC. Scale bar, 5 µm.

**Figure 5:**
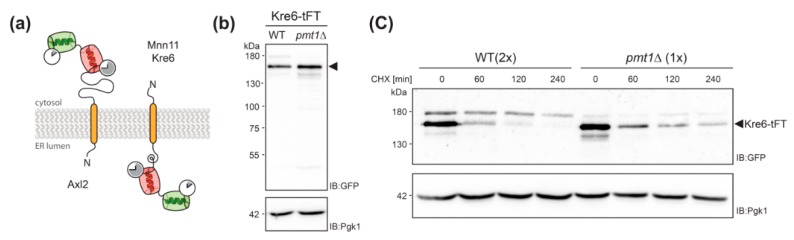
Analyses of Kre6-tFT protein. (**a**) Topology model of Axl2, Kre6 and Mnn11 depicting the orientation of the tFT timer for type I and type II transmembrane proteins. (**b**,**c**) Cell lysates from wild-type (WT; WT Kre6-tFT) and *pmt1*Δ (EZY91) cells expressing Kre6-tFT were resolved on 8% polyacrylamide gels and subjected to Western blot analysis using anti-GFP antibodies. Pgk1 served as a loading control. (**b**) Steady state levels of Kre6-tFT. Cell lysates equivalent to 0.2 OD_600_ units of yeast cells were analyzed. (**c**) Cycloheximide chase analysis as detailed in Materials and Methods. Cell lysates equivalent to 0.2 OD_600_ units (1x; *pmt1*Δ) and 0.4 OD_600_ units (2x; WT) of yeast cells were analyzed, to allow for better comparability. (**b**,**c**) Three forms of Kre6-tFT were detected as previously demonstrated for the native protein [33]. The major band is more abundant at steady state (**b**) and slower degraded (**c**) in mutant *pmt1*Δ. Experiments have been replicated at least two times; representative results are shown.

Kre6 is a type II transmembrane protein involved in the biosynthesis of cell wall β-1,6-glucan [31]. It has been demonstrated previously that a large proportion of the native Kre6 protein is localized in the ER, but a fraction of the protein is also found at the plasma membrane at sites of cell wall growth [32]. Here we observe that the Kre6-tFT fusion protein is present in the ER, but especially in the vacuole (Figure 4a; sfGFP), with the older protein being found exclusively in the vacuole (Figure 4a; mCherry). In mutant *pmt1*Δ, Kre6-tFT is more abundant, and its localization largely corresponds to that in wild-type cells (Figure 4a). The correct localization of other type II transmembrane proteins, such as the Golgi mannosyltransferase Mnn11, is not changed by the C-terminal tFT (Figure 4b and Figure 5a), ruling out that the orientation of the tFT towards the ER lumen does per se alter protein sorting and/or targeting. It was previously shown that the multiple ER chaperon-like proteins contribute to the correct folding and localization of Kre6 [33,34]. Thus, the large C-terminal tFT tag might hamper such interactions and in turn Kre6 ends up in the vacuole.

Next, we monitored abundance of Kre6-tFT in cell lysates of wild-type and *pmt1*Δ cells by SDS-PAGE and Western blot. In addition, stability of the protein was examined by cycloheximide chase analysis as detailed in Material and Methods. As shown in Figure 5b, at steady state significantly more Kre6-tFT protein could be detected in *pmt1*Δ cells when compared to wild-type. Following the addition of the translational inhibitor cycloheximide, Kre6-tFT was readily degraded in wild-type cells. However, loss of Pmt1 substantially stabilized the tFT-fusion protein (Figure 5c) confirming that degradation of the Kre6-tFT protein is negatively affected in absence of Pmt1.

In summary, our data show that the tFT readout allows tracking the impact of *O*-mannosylation not only on the stability of canonical target proteins, but also subtle effects on the degradation of mislocalized and/or misfolded proteins.

## 3. Discussion

In yeast and other eukaryotes, about 20–30% of all gene products are entering the secretory pathway [35]. During or after translocation, these proteins potentially become subject to modification by protein glycosylation, which is a major function of the ER and a major factor contributing to a protein’s cellular fate. A recent proteome-wide screening identified about 290 glycoproteins as targets of PMT-based *O*-mannosylation. *O*-mannosyl glycans were assigned to 68% of all cell wall proteins, 22% of the ER-localized, 18% of the Golgi-resident, 17% of the vacuolar, and 16% of all plasma membrane annotated proteins [7]. To investigate what impact *O*-mannosylation has for a subset of these proteins, we took advantage in this study of the tandem fluorescent timer approach that, in recent years, was successfully applied to monitor changes in protein abundance and turnover on a proteome-wide scale [21,22,36]. We analyzed tFT fusions of 137 proteins mainly of the secretory pathway, the vacuole and the plasma membrane. Due to the C-terminal localization of the tFT, glycosylphosphatidylinositol (GPI)—anchored cell wall proteins, which are major targets of *O*-mannosylation [7], could not be included.

Under the applied conditions, the abundance and stability of the majority of proteins tested was not strikingly affected (supplemental Table S1; Figure 2). Nevertheless, in the three *pmt*Δ mutants, a total of 39 individual proteins was clearly influenced (Figure 2a–c). Among those, Nis1, YJR015W, Mnl2, Sec12, Emp24, Vtc1, Mns1, Prm5 and Pmt3 show negative mCherry/sfGFP Δ-score values, indicating increased protein abundance and/or stability in *pmt1*Δ, *pmt2*Δ and *pmt4*Δ. These proteins are involved in either ER protein quality control (Mnl2, Mns1, Emp24), cell integrity signaling (Prm5), or membrane trafficking (Sec12, Vtc1); pathways that are affected when *O*-mannosylation becomes limiting [10]. The transcription of some of the corresponding genes is enhanced in response to diminished *O*-mannosylation [9]. Thus, the observed effects are most likely part of previously described compensatory mechanisms counteracting *O*-mannosylation defects [9]. Also, transcription of *PMT3* is enhanced in the absence of other Pmts [9]. The *O*-mannosyltransferase Pmt3, a paralog of Pmt2, is found among the most significantly increased/stabilized proteins (Figure 2). It was shown by biochemical means that Pmt3 is only weakly detected in wild-type cells. However, its abundance strongly increases in the absence of the Pmt1-Pmt2 complex. In addition, the formation of alternative complexes between Pmt3 and other PMT-family members was demonstrated in the absence of e.g., Pmt2 [26], which might further account for stabilization and decreased turnover of Pmt3 revealed in this work. Furthermore, in all the three *pmt*Δ mutants, Tsc3 shows positive mCherry/sfGFP Δ-score values, indicating a decrease in stability when *O*-mannosylation is diminished (Figure 2a–c). Interestingly, Tsc3 is involved in the biosynthesis of sphingolipids [37], which are components of membrane domains critical for the trafficking of GPI anchored proteins out of the ER [38]. GPI-anchored proteins are major Pmt substrates [7], and Pmt1-Pmt2 complexes were even suggested as a means to facilitate ER export of these proteins [39]. Thus, our findings point to a coordination between *O*-mannosylation and sphingolipid biosynthesis to ensure proper ER export of GPI-anchored proteins. It will be an intriguing task to unravel that interplay in the future.

In addition to the target proteins which are evenly affected in all *pmt*Δ mutants, we also found candidates which are modified specifically in the *pmt1*Δ, *pmt2*Δ or *pmt4*Δ mutants. Both increased as well as decreased protein abundance/stabilization were found. Some of the proteins, such as Axl2, Kre6 and Van1 (Figure 2c), show similar behavior in mutants *pmt1*Δ and *pmt2*Δ (negative mCherry/sfGFP Δ-scores) which differ from *pmt4*Δ (positive mCherry/sfGFP Δ-score), most likely reflecting varying specificity of Pmt1-Pmt2 and Pmt4 complexes towards their canonical protein substrates [5,40,41]. Furthermore, as shown in Figure 2, more tFT-fusion proteins revealed negative mCherry/sfGFP Δ-score values in mutant *pmt2*Δ and *pmt4*Δ when compared to *pmt1*Δ. For example in mutant *pmt2*Δ, 11 (Jem1, Emp24, Fab1, Tgl4, Vtc1, YNL058C, Ire1, Lam6, Van1, Vth2, YPR063C) and two (Sur1, YCR061W) tFT-fusion proteins with negative and positive Δ-score values (*p*-value < 0.1 and net Δ-score > 0.2) could be detected, indicating that more proteins are stabilized than destabilized when Pmt2 is absent. Only in mutant *pmt2*Δ, proteins important during ER stress conditions, such as Ire1 and Jem1, and for vacuolar sorting, such as Fab1 and Vth2, are among the most abundant/stabilized proteins (Supplemental Table S1; Figure 2c). Our data are in agreement with the previously described role of Pmts, predominantly of Pmt2, in ER protein quality control [14,19,20], and support a specific role of Pmt2 for ER protein homeostasis. Further, our findings substantiate the reported varying impact on Pmt1 and Pmt2 on protein targets [19,20,41], and support distinct roles and/or acceptor specificities of the mannosyltransferases in the Pmt1-Pmt2 complex. Differences of these enzymes are also reflected by the distinct phenotypes of *pmt1*Δ and *pmt2*Δ mutant strains not only in *S. cerevisiae* [8], but also in *Schizosaccharomyces pombe*, where deletion of the Pmt2- but not the Pmt1-orthologue results in lethality [42].

The tFT screen highlighted PMT-substrates whose stability is altered by defects of the PMT machinery. Among the identified proteins, there may be canonical Pmt targets for which *O*-mannosyl glycans are critical to ensure protein stability. As a proof of concept, we identified Axl2, a known Pmt4-specific substrate protein [30], among the major destabilized proteins exclusively in *pmt4*Δ (Figure 2c). Also, the membrane protein sensors Wsc2 and Wsc4 were detected (Figure 2c). Wsc2 destabilization in *pmt*Δ mutants has been reported [11]. Moreover, while addressing the question of whether luminal orientation of the tFT tag affects the localization of type II transmembrane proteins (see above; Figure 5a), fluorescence microscopy of an independent *pmt4*Δ knock out mutant expressing Mnn11-tFT confirmed destabilization of this protein when *O*-mannosylation is decreased (Figure 4b). As mentioned earlier, in many *O*-mannosylated proteins, the glycans are clustered in distinct serine/threonine-rich regions [4]. The Golgi mannosyltransferase Mnn11 [43] depicts such characteristic features: a serine/threonine-rich region, for which *O*-mannosylation has been demonstrated by mass spectrometry [7], separating transmembrane and catalytic domain (Figure 5a).

On the other hand, we also identified proteins that are stabilized when *O*-mannosylation is diminished, which is especially interesting with respect to the aforementioned role of *O*-mannosyl glycans added by the Pmt1-Pmt2 complex for ER protein quality control and protein degradation [14]. From the candidates that were significantly stabilized in the absence of Pmt1 and Pmt2, Kre6-tFT was analyzed in more detail since the mCherry/sfGFP and sfGFP intensity Δ-score values indicated increased stability and abundance, respectively, suggesting impaired degradation (Figure 2a,b). Stabilization could be confirmed by biochemical means for the Kre6-tFT. However, it was found to mislocalize to the vacuole in wild-type and *pmt1*Δ cells (Figure 4 and Figure 5). It is assumed, that Kre6 is a key protein in β-1,6-glucan synthesis due to its similarity to glycoside hydrolases; however, its precise role is still unknown [31]. Complex folding and maturation of Kre6 has been demonstrated that involves the ER Hsp40 chaperone Kar2 and several chaperone-like proteins such as Rot1 and Keg1, as well as calnexin [33,34]. Although the bulk of the Kre6 protein is localized to the ER, Kre6 cycles between ER and Golgi, and is released to the plasma membrane upon cell polarization [32,33,34]. Interaction with calnexin is important to ensure proper localization of the protein. Degradation of Kre6 is suggested to involve the ER associated degradation pathway, as well as the vacuole [34]. We observed that, in contrast to wild-type protein, Kre6-tFT is predominantly mislocalized to the vacuole (Figure 4a). On Western blot, Kre6-tFT appeared as three protein bands with a pattern highly similar to that of the native mature Kre6 protein [33]. Thus, most likely the tFT-tag is hampering interactions with other components important for proper localization rather than protein folding. There are various possibilities to explain how *O*-mannosylation might contribute to the stabilization of the Kre6-tFT fusion protein. In one attractive scenario, *O*-mannosyl glycans of Kre6-tFT might even enhance the efficiency of vacuolar proteases. With the Kre6-tFT fusion protein, we established a new model to further unravel the unexpected functions of *O*-mannosylation in protein stabilization.

In conclusion, here we present the first high-throughput approach to determining the impact of *O*-mannosylation on protein dynamics. Interestingly, *O*-mannosylation can cause both enhanced and diminished protein abundance and/or stability when compromised. The identified target proteins are a valuable resource to address different underlying molecular mechanisms.

## 4. Materials and Methods

### 4.1. Yeast Strains and Growth Conditions

*S. cerevisiae* strains are listed in Table 2. Strains were grown and transformed under standard conditions. The sequences of oligonucleotides used in this study are available on request.

To de novo delete *PMT1*, *PMT2* and *PMT4* in the selected tFT fusion query strains a DNA fragment with a kanMX6 integration cassette was amplified by PCR on pUG6 [44] (using oligonucleotide pairs 1961/1962, 1963/1964, and 1967/1968, respectively) and transformed by homologous recombination in each corresponding wild-type query according to [45]. Successful recombination of the kanMX6 PCR fragments on each locus was confirmed by PCR using oligonucleotide 1516 in combination with 312 and 1513 for *PMT1* locus, 1518 in combination with 312 and 1515 for *PMT2* locus, and 456a in combination with 312 and 457 for *PMT4* locus.

### 4.2. Tandem Fluorescent Protein Timers Screening and Data Analyses

A subset of query strains expressing tFT-tagged proteins that were shown to be *O*-mannosylated [7] were recorded to localize to the secretory pathway (by classification of translocation according to [35] or inferred from high confidence/manual curated database annotations were selected from the library; Supplemental Table S1) and crossed with MLY201 (*pmt1*Δ), MLY202 (*pmt2*Δ) and MLY204 (*pmt4*Δ) on 1536-colony format plates using the ROTOR HDA pinning robot (Singer Instruments, Somerset, UK) following the SGA methodology described in [24]. In brief, both tFTqueries and MLY201, MLY202, or MLY204 were mated, and the resulting diploids were selected, sporulated, and selected for haploids carrying both the tFT-tagged protein and the *pmt* deletion by sequential pinning followed by selection on appropriate media, as described in [22]. Three technical replicates of each cross were arranged next to each other. Fluorescence intensities of the final colonies were measured after 24 h of growth on synthetic complete medium lacking leucine at 30 °C using Infinite M1000 Pro plate readers equipped with stackers for automated plate loading (Tecan, Männedorf, Switzerland) and custom temperature control chambers. Measurements in mCherry (587/10 nm excitation, 610/10 nm emission, fixed detector gain) and sfGFP (488/10 nm excitation, 510/10 nm emission, fixed detector gain) channels were performed at 400 Hz frequency of the flash lamp, with ten flashes averaged for each measurement.

Failed crosses after haploid selection were excluded from the measurement based on colony size. For background correction, the fluorescence intensities of three negative control colonies arranged next to each sample were subtracted of the average of sample colonies. Fluorescence intensity measurements were log-transformed, and the data for each plate were normalized to the median fluorescence of a reference strain set that was present on every plate as described in [46]. Changes in protein stability between wild-type and mutant were estimated by subtracting the log-ratios of mCherry and sfGFP intensities yielding a Δ-score. A moderated *t*-test implemented in the R package limma was used to compute *p*-values [47]. Plots were generated using the *ggplot2* (v2.2.1) package [48]. Data labels were introduced using functions of the *ggrepel* (v0.7.0) package [49], and heatmaps were generated using the heatmap.2 function of the *gplots* (v3.0.1) package [50].

### 4.3. Fluorescence Flow Cytometry

Cells expressing tFT-fusion proteins were grown to the mid-log phase in synthetic complete medium. The sfGFP and mCherry fluorescence intensities of 20,000 cells were measured using the cell analyzer BD FACSCanto™ (BD Biosciences; Heidelberg, Germany), in collaboration with Flow Cytometry & FACS Core Facility (ZMBH, Heidelberg University; Heidelberg, Germany). To define the age of a certain tFT-fusion protein, a first ratio mCherry/sfGFP was calculated for both wild-type and *pmt*Δ mutant, and the corresponding *p*-value was calculated using Student’s *t*-test and considering the null hypothesis as no showing difference between variances of each dataset. To address the impact of the deletion on protein age, a second ratio was calculated as WT_mCherry/sfGFP_/*pmt*Δ_mCherry/sfGFP_.

### 4.4. Preparation of Cell Extracts And Membranes

To prepare total cell extracts, cells were grown overnight in synthetic complete medium [51] at 30 °C to OD-1, resuspended in breaking buffer (50 mM Tris-Cl, pH 7.4, 5 mM MgCl_2_) supplemented with protease inhibitors (1 mM PMSF, 1 mM benzamidine, 0.25 mM 1-chloro-3-tosylamido-7-amino-2-heptanone, 50 μg/mL of l-1-tosylamido-2-phenylethyl chloromethyl ketone, 10 μg/mL of antipain, 1 μg/mL of leupeptin, and 1 μg/mL of pepstatin), and broken with glass beads. Cell debris was removed by centrifugation at 1500 × *g*_av_ for 5 min at 4 °C.

Total membranes were prepared by centrifugation (20,000 × *g*_av_ for 60 min, 4 °C) of total cell extracts. Membrane pellets were resuspended in membrane buffer (50 mM Tris-Cl, pH 7.4, 5 mM MgCl_2_, 15% glycerol) supplemented with protease inhibitors (as described above).

### 4.5. Cycloheximide Chase Experiments

Cycloheximide chase experiments were performed as described in [52]. In brief, wild-type and *pmt1*Δ cells expressing Kre6-tFT (strains WT Kre6-tTF and EZY91 respectively) were grown overnight in synthetic complete medium at 30 °C to OD-1. Cells were initially sampled as time point zero, and cycloheximide was immediately added to a final concentration of 100 µM. Equal amount of cells were sampled at the following time points: 60, 120, and 240 min. After sampling, the chase was stopped at each time point by adding NaN_3_ to a final concentration of 20 mM, and cells were kept on ice until the last sample was collected. Total cell extracts were prepared from cells sampled at each time point as described above.

### 4.6. Western Blot Analyses

Protein samples were incubated in Laemmli buffer either at 70 °C for 10 min (for total cell extracts) or at 50 °C for 10 min (for membranes), resolved in glycine SDS-polyacrylamide gels (8% polyacrylamide), transferred to nitrocellulose, and visualized by enhanced chemiluminescence using the Amersham Biosciences ECL system (GE Healthcare; Munich, Germany). Blots were incubated with the primary antibodies anti-GFP (dilution 1:5000, #ab13970; Abcam, Cambridge, UK) or anti-HA (dilution 1:10,000, #MMS-101R; Covance, Princeton, NJ, USA), and peroxidase-conjugated anti-rabbit (dilution 1:10,000; #A6154; Sigma-Aldrich, St. Louis, MO, USA) or anti-mouse (dilution 1:10,000; #A9044; Sigma-Aldrich) secondary antibodies respectively. For the detection of loading controls, the same blots were incubated with either anti-Sec61 (for membranes, dilution 1:2500; gift from Karin Römisch) or anti-Pgk1 (for total cell extracts, dilution 1:5000, #A6457 Thermo Fischer, Waltham, MA, USA) as primary antibodies followed by either anti-rabbit or anti-mouse as secondary antibodies.

### 4.7. Microscopy

Cells expressing tFT-fusion proteins were grown to the mid-log phase in synthetic complete medium. Prior to imaging, cells were treated with the vital dye 7-amino-4-chloromethylcoumarin (CMAC, #C2110 Thermo Fischer) to a concentration of 10 µM to stain the lumen of yeast vacuoles. After 15 min incubation at 30 °C, cells were washed once and resuspended in fresh medium.

Single plane images were acquired on a Delta Vision Elite system (Applied Precision, Issaquah, WA, USA) consisting of an inverted epifluorescence microscope (IX71; Olympus, Tokio, Japan) equipped with an LED light engine (SpectraX, Lumencor, Beaverton, OR, USA), 390/18-, 475/28- and 575/25-nm excitation and 435/48-, 525/50- and 624/40-nm emission filters (Semrock, Rochester, NY, USA), a dual-band beam splitter 89021 (Chroma Technology, Bellows Falls, VT, USA), using either a 100× NA 1.4 UPlanSApo or a 60× NA 1.42 Plan ApoN oil immersion objective (Olympus), an sCMOS camera (pco.edge 4.2, PCO), and a motorized stage contained in a temperature-controlled chamber. Image processing and quality control were performed using ImageJ. sfGFP, as well as mCherry, images obtained for wild-type and corresponding *pmt*Δ mutant were processed in the same manner.

## Figures and Tables

**Table 1:** Fluorescence flow cytometry of selected candidate strains. Fluorescence intensities of sfGFP and mCherry were measured by flow cytometry in mutants EZY107 (*pmt4*Δ, Axl2-tFT), EZY91 (*pmt1*Δ, Kre6-tFT), EZY96 (*pmt1*Δ, Vrg4-tFT), EZY106 (*pmt2*Δ, YNL058C-tFT) and the corresponding wild-type strains. Intensity ratios were calculated as detailed in Materials and Methods. Ratios > 1 and < 1 are indicative for protein destabilization and stabilization, respectively. Mean ± SD values of three measurements are shown. Indicated *p*-values were calculated using Student’s *t*-test.

*pmt*Δ Mutant	tFT-Fusion Protein	WT_mCherry/sfGFP_/*pmt*Δ_mCherry/sfGFP_ ± SD	*p*-Value
*pmt4*Δ	Axl2	1.118 ± 0.033	0.088
*pmt1*Δ	Kre6	0.842 ± 0.027	0.037
*pmt1*Δ	Vrg4	0.873 ± 0.040	0.047
*pmt2*Δ	YNL058C	0.787± 0.042	0.010

**Table 2:** *S. cerevisiae* strains used in this study.

Strain	Genotype	Source
BY4741	MATa his3Δ1 leu2Δ0 met15Δ0 ura3Δ0	Euroscarf
MLY201	BY4741 except *pmt1Δ*::*KANMX6*	This study
MLY202	BY4741 except *pmt2Δ*::*KANMX6*	This study
MLY204	BY4741 except *pmt4Δ*::*KANMX6*	This study
YMaM330	MATα *can1Δ::STE2pr-SpHIS5 lyp1Δ::STE3pr-LEU2 his3Δ1 leu2Δ0*::*GAL1pr-I-SCEI-natNT2 ura3Δ0*	[22]
WT Kre6-tFT	YMaM330 except *KRE6::mCherry-sfGFP*	[22]
EZY91	WT Kre6-tFT except *pmt1Δ*::*KANMX6*	This study
WT Vrg4-tFT	YMaM330 except *VRG4::mCherry-sfGFP*	[22]
EYZ96	WT Vrg4-tFT except *pmt1Δ*::*KANMX6*	This study
WT Axl2-tFT	YMaM330 except *AXL2::mCherry-sfGFP*	[22]
EZY107	WT Axl2-tFT except *pmt4*Δ	This study
WT YNL058C-tFT	YMaM330 except *YNL058C::mCherry-sfGFP*	[22]
EZY106	WT YNL058C-tFT except *pmt2*Δ	This study
WT Mnn11-tFT	YMaM330 except *MNN11::mCherry-sfGFP*	[22]
EZY109	WT Mnn11-tFT except *pmt4*Δ	This study
MGY69	*AXL2::HA*	[30]
MGY72	*AXL2::HA* except *pmt4*Δ	[30]

## Supplementary Materials

The following are available online at http://www.mdpi.com/1420-3049/23/10/2622/s1, Table S1. List of tFT-fusion proteins and results from automated microscopic fluorescence analysis, Figure S1. Dynamics of tFT-fusion proteins in different *pmt*Δ strains.
