# Supplementary material for: Monitoring Protein Dynamics in Protein O-Mannosyltransferase Mutants In Vivo by Tandem Fluorescent Protein Timers
Source: Molecules. 2018 Oct 12;23(10):2622. doi: 10.3390/molecules23102622 (PMC6222916; doi:10.3390/molecules23102622)
Supplement: Supplementary file 1 [file molecules-23-02622-s001.zip › Supplemental Figure S1.pdf]

Supplemental Figure S1

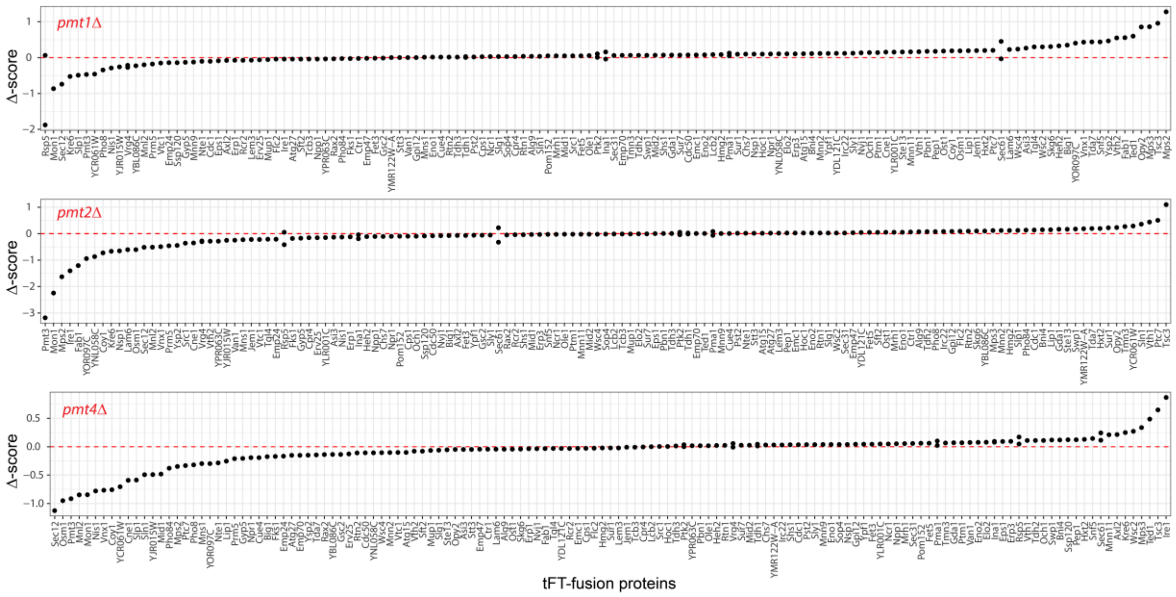

Figure S1. Dynamics of tFT-fusion proteins in different *pmtΔ* strains. Differences in fluorescence intensities of mCherry and sfGFP are compared between mutant and wildtype strain (Δ-score) and summarized in an ordered dot plot. A negative Δ-score indicates a prolonged lifetime of the tFT-fusion protein in the mutant compared to the wildtype, while a positive Δ-score indicates destabilizing effects and a higher turnover of protein. Measurements were performed by high throughput fluorescence microscopy of triplicates. Proteins with multiple results were spotted on multiple plates for internal control. Results are shown as a measure of variance between experiments.
